# The La Region of Foot-and-Mouth Disease Virus: Essential for L Protein Cellular Distribution but Not Functional Activity

**DOI:** 10.3390/ijms27062893

**Published:** 2026-03-23

**Authors:** Mengting Cai, Hong Yuan, Tao Wang, Yuanfang Fu, Huifang Bao, Pinghua Li, Han Weng, Junfang Zhao, Kun Li, Pu Sun, Xueqing Ma, Zhixun Zhao, Jing Zhang, Yimei Cao, Dong Li, Zengjun Lu, Xingwen Bai

**Affiliations:** 1 State Key Laboratory of Animal Disease Control and Prevention, College of Veterinary Medicine, Lanzhou University, Lanzhou Veterinary Research Institute, Chinese Academy of Agricultural Sciences, Lanzhou 730000, China; 2 Gansu Province Research Center for Basic Disciplines of Pathogen Biology, Lanzhou 730046, China

**Keywords:** FMDV, L protein, La region, encoding function

## Abstract

Foot-and-mouth disease virus (FMDV) is a highly contagious picornavirus that affects cloven-hoofed animals and carries significant economic implications for the global livestock industry. FMDV features two Leader (L) protein isoforms, Lab and Lb, differing at their amino termini by 28 amino acids (La region). Currently, the activity of La protein sequences has not been investigated. To address this issue, the comparison study of biological and functional roles of Lab and Lb was performed as the La region alone did not independently perform protein function. We found that Lab and Lb significantly regulated FMDV replication and pathogenicity, and their coexistence afforded optimal FMDV properties. Subsequently, we observed that both L isoforms cleaved eukaryotic translation initiation factor 4G (eIF4G) I, suppressed type I and type III interferon (IFN) expression, and exhibited marked cytotoxicity, indicating that they were all key components in FMDV’s antagonism of host antiviral defenses. Finally, the subcellular distribution of Lab and Lb was detected. Despite dual localization in cytoplasmic and nuclear compartments, both isoforms displayed different spatial distribution patterns, and Lb induced more pronounced morphological changes to host cells than Lab. Furthermore, bioinformatics predicted that the La region might contain a non-classical secretory signal peptide, potentially facilitating Lab distribution to the cell membrane or extracellular space. Collectively, the primary encoding role of La region was to control the intracellular distribution of L protein, as opposed to regulating its functional activity. This study may help to deepen our understanding of why FMDV encoded two isoforms of L protein.

## 1. Introduction

FMDV, an *aphthovirus* in the family *Picornaviridae*, is the etiological agent of foot-and-mouth disease (FMD), a highly contagious and economically devastating disease affecting livestock globally [1,2]. The FMDV Leader (L) protein, a papain-like cysteine protease, is a critical virulence factor and plays a pivotal role in the FMDV pathogenicity [3,4,5]. The highly variable L gene is positioned directly downstream of the FMDV internal ribosome entry site (IRES). Within its N-terminal region (the La region), two conserved, in-frame initiation codons (Lab AUG and Lb AUG) are spaced 84 nucleotides apart. Through the utilization of these dual start sites, FMDV encodes two distinct protease isoforms: Lab, initiated at the first AUG, and the truncated Lb, which initiates at the second AUG [6,7,8].

During FMDV infection, both Lab and Lb isoforms are co-synthesized, with Lb being the predominant isoform [9,10]. Therefore, the majority of reported functional studies on L protein have concentrated on characterizing the properties of Lb, while the biological roles of Lab remain poorly explored. Definitive studies have confirmed that two isoforms of L protein could process the L/P1 junction to release themselves from the polyprotein and cleave the cellular translation initiation factor eIF4GI [11,12]. Additionally, the Lb isoform also regulates the key molecules of the IFN pathway and targets multiple host proteins to antagonize antiviral innate immunity [3,13,14,15,16,17,18]. Notably, all currently known active sites of L protein are located within the Lb region, implying that the Lab likely shares similar functions to Lb. This raises a fundamental biological question: Why does FMDV maintain two seemingly redundant L isoforms?

The Lab or Lb coding sequence is not essential for FMDV survival but affects FMDV replication and virulence. Studies have shown that complete deletion of the entire L coding region, which abolishes both Lab and Lb isoforms, is lethal for FMDV O1Kaufbeuren [19]. However, deletion of the Lb coding sequence, which also prevents Lab and Lb translations, does not impair viral viability in strains such as O1Kaufbeuren, A12, or A24. Furthermore, deletion of the Lb region results in largely unchanged replication of the O1Kaufbeuren virus in BHK-21 cells but decreased replication in pBTY cells. In the case of strain A12, the same deletion reduces viral replication, weakens the ability to suppress IFN expression, and attenuates the pathogenesis in pigs and cattle [14,20,21,22]. In addition, deletion of the La region in O1Kaufbeuren, which retains Lb translation, yields a viable virus with near-normal replication in BHK-21 cells [19]. Our previous study indicates that mutating the Lb AUG of strain O/HN/CHN/93 to AUC or AAA, which allows only Lab translation, does not adversely alter viral growth rates in BHK-21 cells, but leads to smaller plaques and slower replication of mutant viruses in PK-15 cells [23]. From these results, it can be concluded that: (1) the deletion of either the entire Lab coding region or Lb region disrupts the translation of both Lb and Lab, but they lead to distinct viral viability; (2) the independent translation of Lb or Lab isoform attenuates FMDV; (3) phenotypic effects of the same L-region deletion are strain-specific in FMDV. In summary, the influence of L coding region on FMDV characteristics is complicated and multifaceted. It seems that FMDV exhibits greater fitness with improved replication and virulence when dual Lab and Lb isoforms are expressed, further highlighting a crucial encoding role of the La region.

In this study, we generated four FMDV strains encoding the defined isoforms of L protein. Additionally, recombinant plasmids that contained the corresponding L gene sequences of these viruses were also constructed. Using both in vivo and in vitro approaches, we aimed to elucidate the specific functional contributions of the La region to L protein activity by investigating how the Lab and Lb isoforms regulated FMDV biological characteristics, as well as by examining their molecular functions and subcellular distribution.

## 2. Results

### 2.1. Rescue and Identification of FMDV Mutants Translating Different Isoforms of L Protein

**Figure 1:**
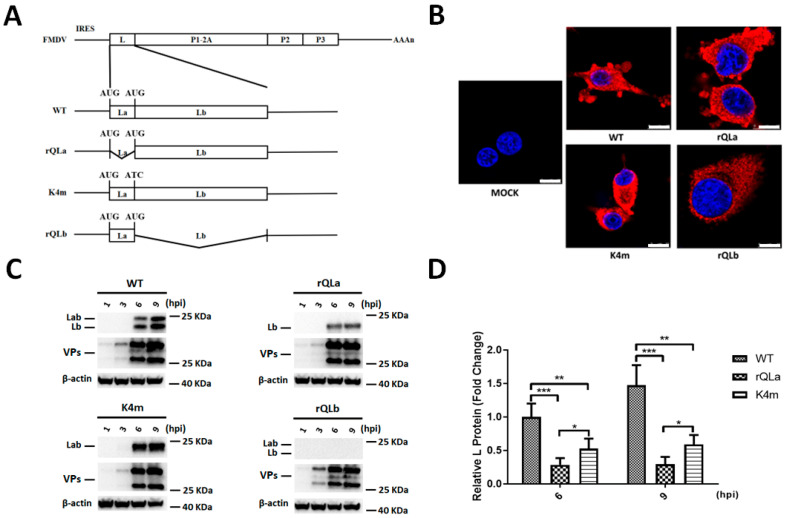
Structure and identification of rescued FMDV mutants. (**A**) The full-length cDNA structure of the modified FMDVs encoding the intended L protein. Deletion of the La region between the two initiation AUGs resulted in the obtainment of rQLa; deletion of the Lb region resulted in the production of rQLb; modification of the Lb initiation AUG to AUC resulted in the generation of K4m. (**B**) BHK-21 cells were infected with rescued viruses at 10 MOI for 4 h. IFA was performed, and FMDV 3A protein was detected using antibodies MAb 3A24 and Cy3-conjugated anti-Mouse IgG (H + L) (red). Scale bar, 10 μm. (**C**) BHK-21 cells were infected with rescue viruses at 1 multiplicity of infection (MOI), and samples were collected at 1, 3, 6, and 9 h post-infection (hpi). The L protein species were analyzed by Western blot. (**D**) The total synthesis of L isoforms was analyzed by densitometry and quantified relative to the L protein levels in WT-infected cells. Statistical significance was denoted as follows: * *p* < 0.05; ** *p* < 0.01; *** *p* < 0.001.

The infectious cDNA plasmid derivatives pOFS-QLb and pOFS-QLa were constructed by replacing the L gene in the full-length cDNA pOFS for FMDV O/HN/CHN/93 with the La or Lb gene, respectively. In total, four full-length cDNA plasmids, pOFS, pOFS-QLa, pLab4m, and pOFS-QLb, as shown in Figure 1A, were linearized by Not I and transfected into BSR/T7 cells. The transfected cell samples were harvested and serially passaged in BHK-21 cells until the cytopathic effect (CPE) appeared. Four viable viruses were finally yielded, namely WT, rQLa, K4m, and rQLb. The L gene of rescued viruses was sequenced, and the results confirmed that the engineered modification of L gene in each FMDV mutant was maintained, demonstrating the generation of four distinct FMDV strains carrying the intended L gene variants. Additionally, the rescued viruses were identified by indirect immunofluorescence assay (IFA) using the primary antibody MAb 3A24 and a Cy3-conjugated secondary antibody (CWBIO, Jiangsu, China). As shown in Figure 1B, all the virus-infected BHK-21 cells exhibited red fluorescence when stained with MAb 3A24, while the normal BHK-21 cells showed no fluorescence, indicating that the rescued viruses were infectious FMDV strains expressing 3A protein.

In order to detect the isoforms of L protein encoded by the rescue viruses, the synthesis of L protein during mutant FMDV infection was examined by Western blot using rabbit polyclonal serum against L protein. As expected, the WT virus synthesized both Lab and Lb, whereas rQLa produced only Lb, and K4m generated solely Lab. Neither Lab nor Lb was detected in rQLb-infected cells (Figure 1C). Collectively, these findings indicated that the engineered FMDV mutants produced specific L protein isoforms as intended. In addition, total L protein production among the four rescued viruses was quantified through the previously described methods [23]. The total synthesis of L protein products by rQLa or K4m was significantly lower than that of the WT virus. Notably, rQLa produced particularly less Lb than the Lab generated by K4m, suggesting the critical involvement of La region in FMDV translation (Figure 1D).

### 2.2. Effect of Both L Isoforms on the Biological Characteristics of FMDV Mutants

**Figure 2:**
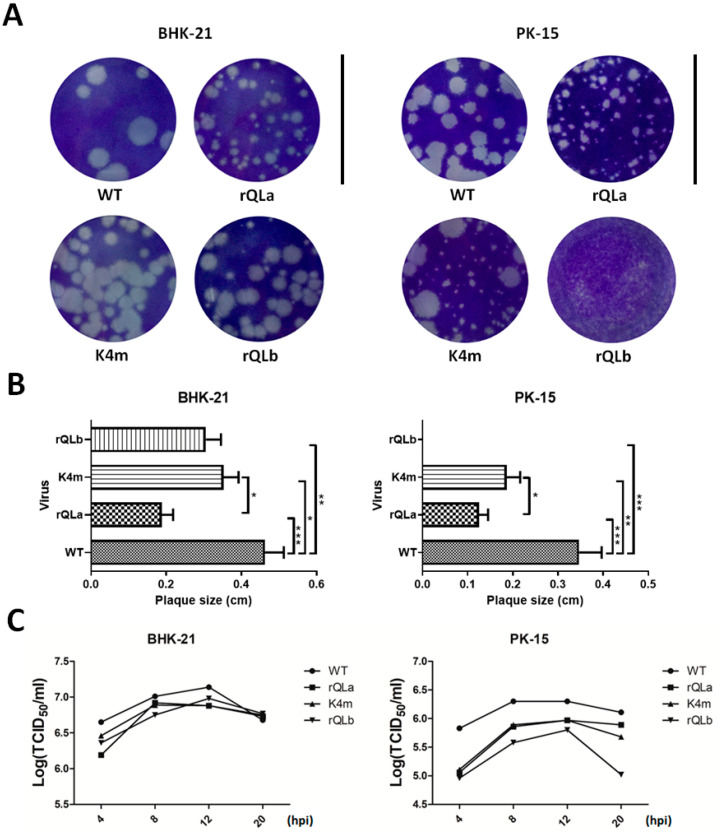
Plaque morphology and one-step growth curve of FMDV variants expressing specific L isoforms in BHK-21 and PK-15 cells. (**A**) BHK-21 or PK-15 cells were incubated with the diluted WT and mutant FMDVs, and the plaque-forming assay was performed. The plaque morphology was observed for each virus at 45 hpi. Scale bar, 3.5 cm. (**B**) The size of about 100 plaques was analyzed for each virus. Data represent the mean ± SD of three independent experiments (* *p* < 0.05; ** *p* < 0.01; *** *p* < 0.001). (**C**) BHK-21 or PK-15 cells were infected with FMDV at 1 MOI for 4, 8, 12, and 20 h. Cell samples were harvested, and virus titers were determined using 50% tissue culture infective dose (TCID_50_) assay. Data were presented as mean values from three independent experiments.

The plaque phenotype of FMDV primarily reflects viral replication efficiency, virulence, and host cellular tropism, while the one-step growth curve of FMDV specifically characterizes viral replication capability. Using two distinct cell lines, interferon-deficient BHK-21 cells with impaired antiviral responses and PK-15 cells possessing functional interferon signaling that effectively suppressed FMDV replication, we systematically compared the effects of both L isoforms on FMDV plaque phenotype (Figure 2A,B) and replication kinetics (Figure 2C). The results showed that on BHK-21 cells, rQLa, K4m, and rQLb mutants were able to form plaques but produced significantly smaller plaques than the WT FMDV. Notably, K4m and rQLb formed larger plaques than rQLa, suggesting that L isoforms were not essential for FMDV plaque formation on BHK-21 cells, and the La coding region contributed more substantially to viral replication than the Lb region in BHK-21 cells. In contrast, on PK-15 cells, both rQLa and K4m, expressing either Lb or Lab, had smaller plaque sizes compared to the WT virus, whereas rQLb, which lacked all L protein isoforms, failed to form visible plaques. These demonstrated that the presence of any L protein isoform was required for FMDV plaque formation on PK-15 cells. Moreover, rQLa formed smaller plaques than K4m, aligning with the phenotype observed on BHK-21 cells.

The one-step growth curve indicated that K4m, rQLa, and rQLb had slightly lower replication titers than the WT virus in BHK-21 cells. In comparison, K4m, rQLa, and rQLb grew more slowly relative to the WT virus in PK-15 cells, with rQLb exhibiting the most pronounced replication defect. These findings were consistent with the plaque phenotype observations, confirming that FMDV variants expressing either L isoform had higher infectivity than those lacking both isoforms of L protein in less permissive PK-15 cells.

### 2.3. Pathogenicity in Suckling Mice of FMDV Mutants Encoding the Intended L Protein

**Figure 3:**
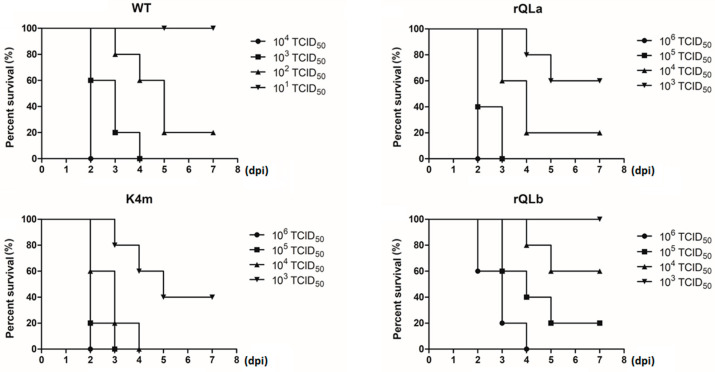
Effect of two L isoforms on FMDV virulence in suckling mice. Suckling mice were inoculated with four different doses of WT and mutant viruses. Survival of suckling mice was tracked, and the survival percentage was calculated daily for 7 days post-infection. Survivors were humanely euthanized at the experimental endpoint.

The effect of Lab and Lb isoforms on FMDV pathogenicity was further examined. Suckling mice were inoculated with serial dilutions of WT virus (10^4^, 10^3^, 10^2^, and 10^1^ TCID_50_/200 μL) or mutant viruses (10^6^, 10^5^, 10^4^, and 10^3^ TCID_50_/200 μL), and the survival rate was calculated for 7 consecutive days (Figure 3). All mice died in the WT group when inoculated with a dose of 10^3^ TCID_50_, while this happened in the mutant group at the following doses: 10^5^ TCID_50_ for the rQLa group, 10^4^ TCID_50_ for the K4m group, and 10^6^ TCID_50_ for the rQLb group. Additionally, the survival rates decreased with the increasing viral doses. At 10^3^ TCID_50_, only WT virus induced complete lethality, and both WT and K4m viruses achieved 100% mortality at 10^4^ TCID_50_. Remarkably, even at a high inoculum dose of 10^5^ TCID_50_, 20% of mice infected with rQLb still survived. Together, the above results demonstrated that both Lab and Lb contributed to FMDV virulence, and their combined presence produced maximal pathogenicity.

### 2.4. Function of L Isoforms on eIF4GI Cleavage

**Figure 4:**
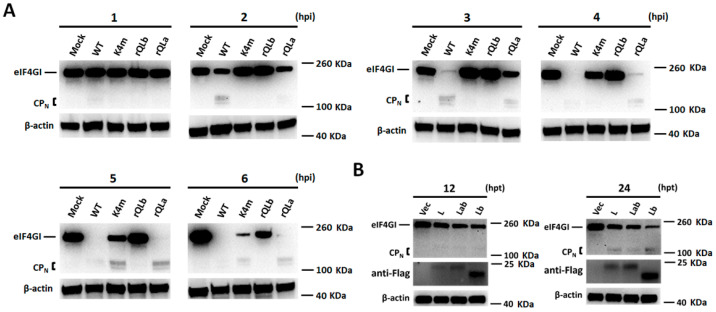
eIF4GI cleavage mediated by Lab and Lb proteins. (**A**) PK-15 cells were infected with WT and mutant FMDVs at 1 MOI. Cell samples were collected at 1, 2, 3, 4, 5, and 6 hpi, and the levels of eIF4GI were detected by Western blot. (**B**) PK-15 cells seeded in 12-well plates were transfected with 1 μg/well of either empty vector Flag, Flag-L, Flag-Lab, or Flag-Lb plasmid. Cell samples were harvested at 12 and 24 h post-transfection and subjected to Western blot analysis to quantify eIF4GI protein levels.

Beyond regulating the biological characteristics of FMDV, L protein also functions as a multifunctional antagonist of host cell defenses. Firstly, we assessed the cleavage of host eIF4GI mediated by Lab and Lb synthesized during FMDV infection. As shown in Figure 4A, the WT infection induced almost complete eIF4GI cleavage by 3 hpi, while rQLa required 5 h for full cleavage. A small proportion of eIF4GI was still retained in K4m-infected cells at 6 hpi, whereas rQLb infection preserved a large amount of intact eIF4GI at this time point. Based on the above results, it was concluded that FMDV mutants encoding either L isoform were able to cleave eIF4GI, though with reduced efficiency compared to the WT virus. In contrast, the FMDV mutant lacking functional L protein nearly abolished eIF4GI cleavage, suggesting that both Lab and Lb were essential for efficient eIF4GI cleavage, and their deficiency correlated with a markedly reduced capacity of FMDV to shut down the translational initiation of the host cells.

Moreover, eIF4GI cleavage was investigated in cells transfected with Flag-tagged plasmids Flag-L, Flag-Lab, and Flag-Lb, respectively (Figure 4B). Western blot analysis confirmed that all overexpression plasmids were able to express L protein, among which Flag-Lb and Flag-Lab plasmids properly expressed their designated L isoform, while Flag-L exclusively expressed Lab protein. Both overexpressed Lb and Lab were able to cleave eIF4GI, but the cleavage levels in Lb-overexpressed cells were higher than those in Lab-overexpressed cells, which may correlate with the correspondingly higher Lb protein levels compared to Lab. In conclusion, both isoforms of L protein expressed by overexpression plasmids exhibited eIF4GI cleavage activity.

### 2.5. Type I and Type III IFN Expression Levels in the Presence of Two Isoforms of L Protein

**Figure 5:**
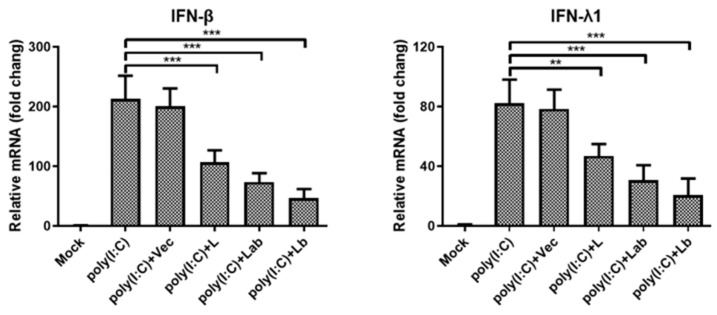
Regulation of IFN expression by two L isoforms. PK-15 cells seeded in 12-well plates were transfected with poly(I:C) (1 ug/well) for 12 h, followed by the transfection of empty vector Flag, Flag-L, Flag-Lb, or Flag-Lab plasmid (1 μg/well) for 24 h. Then, samples were collected, and RT-qPCR was performed to examine the mRNA levels of IFN-β and IFN-λ1. Data represent the mean ± SD of three independent experiments. Statistical significance was denoted as follows: ** *p* < 0.01; *** *p* < 0.001.

In addition to eIF4GI cleavage, L protein counteracts host antiviral defenses by suppressing the innate immune response. To further characterize this function, we determined the regulatory effects of distinct L isoforms on type I and type III IFN expression. Given that multiple FMDV proteins suppressed host IFN expression through diverse mechanisms, we focused specifically on dissecting the individual contributions of L isoforms to IFN suppression using targeted plasmid overexpression (Figure 5). Quantitative analysis revealed that stimulation with poly(I:C) (Sigma) significantly upregulated mRNA levels of IFN-β and IFN-λ1 in PK-15 cells. These IFN inductions were strongly suppressed by the overexpression of Lb or Lab protein, supporting that both isoforms of L protein inhibited IFN expression in host cells.

### 2.6. Modulation of Cytotoxicity by Two L Protein Isoforms

**Figure 6:**
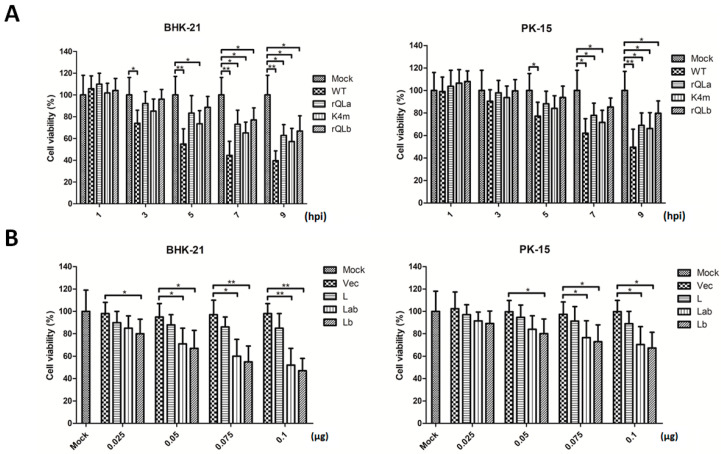
Cytotoxic effects of Lab and Lb proteins in host cells. (**A**) BHK-21or PK-15 cells in 96-well plates were infected with FMDV mutants at a dose of 1 MOI, and cell viability at 1, 3, 5, 7, and 9 hpi was measured by MTS assay. (**B**) BHK-21or PK-15 cells were transfected with increasing doses of empty vector Flag, Flag-L, Flag-Lb, or Flag-Lab plasmid (0.025, 0.05, 0.1, or 0.2 μg/well) for 24 h. Cell viability was then evaluated. Data are expressed as mean ± SD of three independent experiments. Statistical significance was denoted as follows: * *p* < 0.05; ** *p* < 0.01.

Finally, the toxicity of different isoforms of L protein derived from either FMDV mutants (Figure 6A) or through in vitro overexpression (Figure 6B) was evaluated in host cells. Infection with FMDV mutants exhibited significant cytotoxicity in both BHK-21 and PK-15 cells, with more pronounced effects observed in BHK-21 cells. Furthermore, the viral infection led to a time-dependent reduction in cell viability, correlating with the development of characteristic CPE. Comparative analysis revealed that rQLa and K4m mutants displayed reduced cytotoxicity relative to the WT virus, yet remained more toxic than the rQb mutant. The cytotoxic effects of overexpressed Lab and Lb isoforms were next evaluated. Both protein isoforms exhibited dose-dependent cytotoxicity in BHK-21 and PK-15 cells, with significantly reduced cell viability observed at higher plasmid transfection doses compared to empty vector control. Additionally, PK-15 cells were more resistant to L protein-induced cytotoxicity than BHK-21 cells, consistent with their relative susceptibility to FMDV infection. These findings further confirmed that both Lab and Lb proteins functioned as virulence factors of FMDV.

### 2.7. Subcellular Distribution of Different Isoforms of L Protein During FMDV Infection

**Figure 7:**
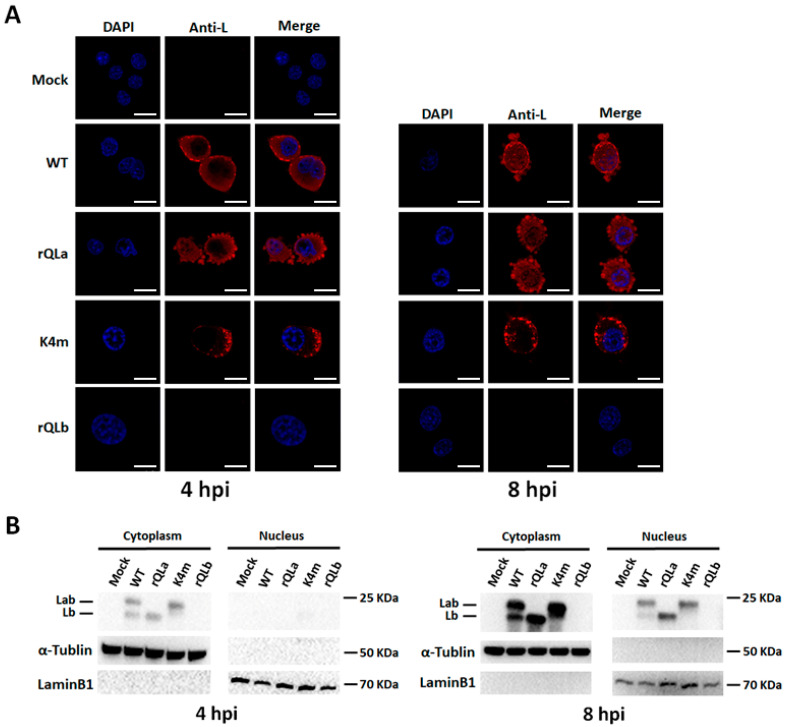
Subcellular localization of Lab and Lb produced by FMDV mutants. (**A**) BHK-21 cells were infected with FMDV mutants at a dose of 1 MOI, and the subcellular distribution of L protein at 4 and 8 hpi was analyzed by IFA using the rabbit polyclonal serum against L protein (red), with DAPI-stained nuclei (blue). Scale bar,10 μm. (**B**) BHK-21 cells were infected with FMDV mutants at a dose of 1 MOI, and samples were collected at 4 and 8 hpi. Cytoplasmic and nuclear extracts were prepared, followed by Western blot analysis of L protein levels. Lamin B1 and α-tubulin were used as nuclear and cytoplasmic fraction controls, respectively.

The subcellular distribution of a protein critically influences its function. To investigate potential functional differences between the two L isoforms, their intracellular distribution was analyzed. Firstly, IFA was performed with an L-specific antibody after the infection of FMDV mutants. As shown in Figure 7A, no immunofluorescence was detected in either negative control cells or rQLb-infected cells; in WT-infected cells, the red immunofluorescence was observed in both cytoplasm and nucleus, with a patchy accumulation near the cell membrane and uniform punctate distribution throughout the cytoplasm and nucleus; in rQLa-infected cells, the immunofluorescence appeared as puncta evenly distributed in the cytoplasm and nucleus; in K4m-infected cells, the majority of immunofluorescence formed aggregated patches adjacent to the cell membrane, while a weaker punctate signal was present in the cytoplasm and nucleus. Moreover, at 8 hpi, cells infected with WT or rQLa, particularly rQLa, displayed substantial nuclear deformation, abundant cytoplasmic vesiculation, and extensive membrane protrusion formation. In contrast, K4m-infected cells maintained relatively normal nuclear, cytoplasmic, and membrane morphology. These observations suggested that the Lb protein disrupted cellular architecture more severely and induced greater cytopathic effects than the Lab isoform.

The intracellular distribution of Lab and Lb was further characterized by the nuclear/cytosol fractionation assay (Figure 7B). Results revealed that at 4 hpi, Lab and Lb were exclusively detected in the cytoplasm of cells infected with WT, rQLa, or K4m, but by 8 hpi, both isoforms were detected in the nucleus and cytoplasm. This temporal shift suggested that the L protein may be initially synthesized in the cytoplasm and subsequently translocated to the nucleus. As expected, no L protein was detected in either negative control or rQLb-infected cells.

### 2.8. Intracellular Distribution of Overexpressed Lab and Lb In Vitro

**Figure 8:**
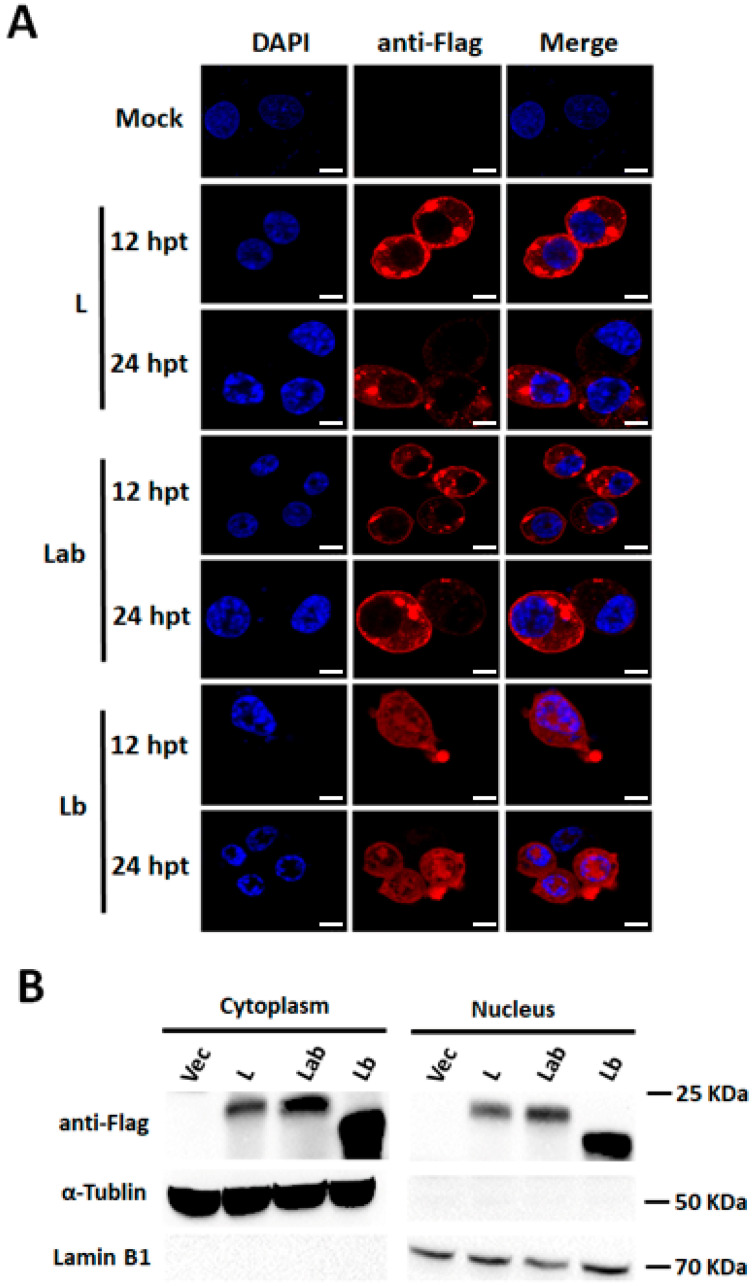
Intracellular distribution of Lab and Lb overexpressed by recombinant plasmids. (**A**) BHK-21 cells were transfected with 1 μg/well of the empty vector Flag, Flag-L, Flag-Lb, or Flag-Lab plasmid for 12 and 24 h, respectively. IFA was performed using the mouse monoclonal antibody against Flag (red), with nuclei counterstained by DAPI (blue). Scale bar, 10 μm. (**B**) Following 24 h transfection with the same plasmids (1 μg/well), cytoplasmic and nuclear extracts were isolated. L protein levels were analyzed by Western blot.

The intracellular distribution of overexpressed Lab and Lb in vitro was also determined (Figure 8A). Interestingly, IFA results proved that the overexpressed Lab displayed dual cytoplasmic and nuclear localization, with the majority forming membrane-proximal aggregates and a minor fraction distributed as cytoplasmic and nuclear puncta. However, the overexpressed Lb exclusively exhibited a punctate distribution pattern, with immunofluorescence signals evenly dispersed throughout both the cytoplasmic and nuclear fractions. No specific immunofluorescence staining was detected in BHK-21 cells transfected with the empty vector control. Consistent with FMDV infection results, cells overexpressing Lb exhibited more pronounced morphological alterations in nuclear, cytoplasmic, and membrane architecture compared to the Lab-expressing cells, suggesting that Lb exerted a more disruptive role in host cell morphology.

The distribution of overexpressed Lab and Lb was subsequently examined through the nuclear/cytosol fractionation assay (Figure 8B). Both proteins were detected in cytoplasmic and nuclear fractions, consistent with their localization observed during FMDV infection. Thus, Lab and Lb exhibited distinct subcellular distribution patterns and differential effects on host cell morphology, demonstrating the functional attribution of the unique La region.

### 2.9. Bioinformatics Prediction of Full-Length L Protein

To further characterize the full-length L protein, bioinformatics analyses focusing on its signal peptide and subcellular localization were performed. The prediction (Table 1) revealed an absence of classical secretion signal peptides; however, residues 6–20 in the La region were identified as a potential non-classical secretion signal. The high hydrophobicity of this La region might facilitate membrane localization or extracellular secretion through non-classical pathways, which aligned with our findings of Lab protein clustering at the plasma membrane periphery. There was no predicted classical nuclear localization signal (NLS) in the L protein. Instead, a potential non-classical NLS at C-terminal residues 194–201, featuring discontinuous basic residues (K195, K198, R199, and K201), was identified. The spatial distribution of these positively charged basic residues may mediate nuclear entry through electrostatic interactions. Consistent with this prediction, our experimental data demonstrated a nuclear accumulation of both Lab and Lb. In addition to the predicted localization in both the cytoplasm and nucleus, L protein may also be secreted extracellularly. However, there was no predicted transmembrane α-helix domain in the full-length L sequence, suggesting that L protein could not be directly integrated into the cell membrane. To sum up, the comprehensive analysis based on bioinformatic predictions with experimental validations revealed that the unique features of the La protein sequences possibly drove isoform-specific functional differentiation of the L isoforms, thus presenting a highly potential target for future research.

## 3. Discussion

FMDV L gene is positioned downstream of the IRES and at the beginning of the single open reading frame (ORF). Within the L gene, two initiation AUG codons allow for the production of two functional L isoforms. The first AUG encodes the full-length Lab protein, and the second AUG produces a truncated Lb protein, differing by the La region deletion relative to the Lab [37,38,39]. While these isoforms are produced, their shared molecular functions remain largely uncharacterized. This knowledge gap hinders our understanding of the specific contributions of Lab and Lb to FMDV replication and pathogenesis, particularly the biological relevance of the N-terminal La region unique to Lab. To address this issue, we employed reverse genetics to rescue FMDV mutants expressing distinct L isoforms and constructed recombinant plasmids overexpressing the corresponding L proteins.

Our initial assessment focused on how these L isoforms influenced the biological properties of FMDV. We observed that the rQLb mutant, which lacked the Lb region and was thus deficient in both L isoforms, demonstrated significantly impaired replication and failed to form visible plaques on PK-15 cells. Furthermore, its pathogenicity in suckling mice was markedly attenuated relative to the WT virus, establishing the L protein as a critical determinant of FMDV replication and virulence. Conversely, the rQLa, characterized by the deletion of the La region and loss of the Lab isoform, and the K4m, bearing a mutation at the Lb AUG that abolished the Lb isoform, produced observable plaques on PK-15 cells, exhibited significantly higher replication titers, and displayed markedly enhanced pathogenicity in suckling mice in comparison with the rQLb mutant. These results underscored the critical contributions of both Lab and Lb proteins to FMDV replication and virulence. Interestingly, both rQLa and K4m mutants exhibited reduced plaque sizes, lower replication titers, and attenuated pathogenicity when compared to WT. This strongly suggested that the co-expression of Lab and Lb was essential for optimal FMDV replication and virulence. Further analysis elucidated the dual antiviral roles of different L isoforms in antagonizing host cells. We found that both Lab and Lb, synthesized during FMDV infection, not only cleaved eIF4GI but also induced significant host cell toxicity. Consistent with this, in vitro overexpression of both L isoforms similarly cleaved eIF4GI, inhibited type I and type III IFN expression, and caused significant host cell toxicity. These findings highlighted Lab and Lb as crucial effectors of FMDV’s antiviral strategy against host cells.

Finally, we analyzed the subcellular distribution of two L isoforms using IFA, nucleocytoplasmic fractionation, and bioinformatics prediction. Experimental results showed that both Lab and Lb localized to the cytoplasm and nucleus. Notably, Lb exhibited a uniform and extensive distribution throughout these compartments, contrasting with the aggregate localization of Lab around the cell membrane. Further, Lb significantly altered host cell morphology, impacting the nucleus, cytoplasm, and cell membrane more profoundly than Lab. Bioinformatics predictions indicated that the Lab potentially contained a non-classical secretion signal at its N-terminus and a non-classical NLS at its C-terminus, while Lb carried a single NLS at its C-terminus, which may lead to differences in the intracellular distribution of Lab and Lb. It has been reported that upon cellular exposure to stress, dynamic aggregates, such as stress granules (SPs) and lipid droplets (LDs), form within cells as a responsive adaptation to environmental changes, ultimately contributing to cell survival and homeostasis [40,41]. Given that both Lab and Lb were virulence factors of FMDV, the uniform cytoplasmic and nuclear distribution of Lb likely facilitated its broad participation in various intracellular signaling pathways, thereby enabling comprehensive regulation of cellular physiological activities for FMDV proliferation. In contrast, Lab aggregates may function as a reservoir for the L protein, releasing it when required to modulate viral virulence towards cells. However, whether Lab was processed into Lb under specific conditions required further in-depth investigation. Therefore, the differential distribution of Lab and Lb probably conferred complementary or competitive functional roles to these isoforms, promoting cellular multifactoriality and enhancing optimal viral replication within host cells.

We observed that at 6 hpi, the L levels translated from the K4m were markedly higher than those from the rQLa, suggesting that the non-coding function of the La region promoted L protein synthesis by enhancing viral mRNA translation efficiency. Furthermore, the rQLb mutant, which was incapable of translating the L protein, produced substantially larger plaques on BHK-21 cells compared to the rQLa, which expressed the Lb protein. This discrepancy was likely attributed to the regulatory influence of the La gene on FMDV replication. Collectively, these findings indicated that the La region functioned not only through its coding capacity but also via non-coding regulatory mechanisms, a conclusion consistent with prior reports. Previous studies demonstrated that inserting a 57-nt transposon between the two AUGs slowed FMDV replication and attenuated its pathogenicity in cattle. Conversely, deleting the 51-nt sequence between these AUGs did not alter viral replication or virulence [42,43]. To date, however, the detailed molecular mechanisms by which the non-coding functions of the La region regulate FMDV replication and pathogenesis remain elusive. Elucidating the coding functions of the La region, as performed in this study, provides a necessary foundation for our subsequent investigations into these underlying non-coding molecular mechanisms.

## 4. Materials and Methods

### 4.1. Cells, Plasmids and Antibodies

The information on BHK-21, BSR/T7, and PK-15 cells has been described previously [44]. The cells were maintained in Dulbecco’s modified Eagle’s medium (DMEM, Gibco, Carlsbad, CA, USA) supplemented with 10% fetal bovine serum (FBS, Sigma, St. Louis, MO, USA), 100 U/mL penicillin, and 100 μg/mL streptomycin (Gibco) in 5% CO_2_ at 37 °C. The full-length infectious cDNA clone plasmid pOFS and the half-length plasmid pSK-Z123 are constructed and preserved by our laboratory. The plasmid pLab4m, a derivative of pOFS, carries an AUG-to-AUC mutation in the Lb initiation codon and encodes only the Lab protein. Mouse monoclonal antibody MAb 3A24, which recognizes and reacts with FMDV 3A protein, and rabbit polyclonal serum against L protein have been reported previously [23].

### 4.2. Plasmid Construction

The synthesized La or Lb gene (Genewiz, Suzhou, China) was individually cloned into plasmid pSK-Z123 after digestion with Xba I and BamH I. The inserted sequences were confirmed by sequencing. Subsequently, fragments digested by Spe I and Bgl II were cloned between the Spe I and Bgl II sites of plasmid pOFS to construct the full-length cDNA plasmids. The final plasmids with correct sequences were named pOFS-QLb and pOFS-QLa.

The corresponding L gene sequences derived from plasmids pOFS, pLab4m, and pOFS-QLa were separately introduced into the pCMV-flag vector (Beyotime, Shanghai, China) using enzymes Pst I and Xho I. The recombinant plasmids Flag-L, Flag-Lab, and Flag-Lb were sequenced to confirm the inserted gene fragment.

### 4.3. Virus Rescue and Identification

The full-length plasmid was linearized by Not I and subsequently transfected into BSR/T7 cells expressing the T7 RNA polymerase using Lipofectamine™ 2000 (Invitrogen, Carlsbad, CA, USA). Transfected cells were observed until the occurrence of typical FMDV-induced cytopathic effects. The culture supernatant of transfected cells was collected and serially passaged in BHK-21 cells. The rescued virus was confirmed by L gene amplification and sequencing. The titer of virus stocks at passage 7 was quantified by calculating the 50% tissue culture infectious dose per mL (TCID_50_/mL).

### 4.4. Indirect Immunofluorescence Assay

Cells were grown on 20 mm culture dishes (NEST, Wuxi, China) and infected with rescued FMDV at a MOI of 10 or transfected with recombinant plasmid (1 μg/well) for the indicated time. Cells were fixed in 4% paraformaldehyde (Solarbio, Beijing, China) for 30 min, permeabilized with 0.5% Triton X-100 (Sigma-Aldrich, St. Louis, MO, USA) for 15 min, and blocked with 1% bovine serum albumin (BSA) (Sigma-Aldrich) for 1 h. Later, the primary antibody and secondary antibody were incubated, respectively, and the nuclei were stained with 4′,6-diamidino-2-phenylindole (DAPI) (Beyotime). At last, cell samples were analyzed using the laser-scanning confocal microscope (Leica Camera AG, Wetzlar, Germany).

### 4.5. Plaque-Forming Assay

The FMDV was serially diluted 10-fold in serum-free MEM (Gibco). Cells in six-well plates were incubated with the diluted FMDV for 1 h at 37 °C, followed by the addition of 2 mL overlay (1% gum in 2× MEM containing 1% FBS). After incubation at 37 °C for 45 h, the cells were fixed with 50% acetone and 50% methanol and stained with 0.2% crystal violet. Plaque morphology was then analyzed.

### 4.6. One-Step Growth Curve Analysis

Cells were infected with FMDV at an MOI of 1 at 37 °C for 1 h. Cell samples were harvested at 4, 8, 12, and 20 hpi. Virus titers were determined by TCID_50_ assay, and a one-step growth curve was generated.

### 4.7. Pathogenicity in Suckling Mice

The animal experiments were approved by the Animal Ethics Committee of Lanzhou Veterinary Research Institute (Permit No. LVRIAEC-2018-036). One-day-old suckling mice were randomly divided into five groups. Following serial dilution in serum-free MEM, FMDV was administered via subcutaneous inoculation at varying TCID_50_ doses. A control group was inoculated with an equivalent volume of neutral PBS. The percentage of surviving mice was calculated for one week post-inoculation.

### 4.8. Western Blot Analysis

The collected cell samples were lysed and subjected to 4–20% sodium dodecyl sulfate-polyacrylamide gel electrophoresis (SDS-PAGE). The resolved proteins were electrotransferred to polyvinylidene difluoride membranes (Millipore, Billerica, MA, USA). After blocking in 5% non-fat milk, membranes were probed with the primary antibody and an HRP-conjugated secondary antibody. Finally, protein bands were detected using Western Lightning^®^ Plus ECL (PerkinElmer, Waltham, MA, USA).

### 4.9. Quantitative RT-PCR (RT-qPCR)

Samples were harvested and lysed, followed by total RNA extraction using the RNeasy Mini Kit (Qiagen, Hilden, Germany). Genomic DNA was eliminated by DNase I treatment (Thermo Fisher Scientific, Waltham, MA, USA). RNA was then reverse-transcribed into cDNA using M-MLV Reverse Transcriptase (Takara, Dalian, China). Quantitative PCR was performed on an ABI 7500 Real-Time PCR System (Applied Biosystems, Foster City, CA, USA) with SYBR Green PCR Master Mix (Takara, Dalian, China) using previously published IFN-specific primers [23]. The mRNA levels were quantified in triplicate and normalized to glyceraldehyde-3-phosphate dehydrogenase (GAPDH) expression. Relative transcript levels were calculated using the 2^−ΔΔCT^ method and expressed as fold-changes compared to mock-treated controls.

### 4.10. Cell Viability Measurement

Cells were seeded in 96-well plates and treated with FMDV (1 MOI) or transfected with plasmid DNA (0.125 μg/well). At the indicated time, 10 μL/well of CellTiter 96 Aqueous One Solution Reagent (Promega, Madison, WI, USA) was added to cells to assess cell viability based on 3-(4,5-dimethylthiazol-2-yl)-5-(3-carboxymethoxyphenyl)-2-(4-sulfophenyl)-2H-tetrazolium. Following 3 h incubation at 37 °C, the absorbance was measured at 490 nm using a microplate reader (Bio-Rad).

### 4.11. Nuclear/Cytosol Fractionation Assay

The cells, which were treated by FMDV infection or plasmid transfection, were harvested and fractionated into nuclear and cytosolic components using Nuclear/Cytosol Fractionation Kit (BioVision, Milpitas, CA, USA) according to the recommended protocols.

### 4.12. Statistics Analysis

The results were representative of at least three independent experiments and presented as the mean ± standard deviation of triplicate experiments. Statistical analysis was performed using the unpaired Student’s *t*-test, with significance defined as *p* < 0.05 (*), *p* < 0.01 (**), and *p* < 0.001 (***).

## 5. Conclusions

This study systematically analyzed the functional characteristics of Lab and Lb proteins using both in vivo and in vitro approaches. Although direct confirmation of functional differences between the L isoforms was beyond the scope of this study, several findings strongly implied distinct biological roles. These include: (1) significantly different cellular distribution patterns of Lab and Lb; (2) notable differences in the host cell morphology alterations induced by each protein; (3) bioinformatics prediction of a non-classical secretory signal peptide within the N-terminal region of Lab; and (4) the observation that the coexistence of both L isoforms was most advantageous for FMDV infection. Together, our results unequivocally demonstrated that these two L isoforms were not functionally redundant but rather represent a deliberate strategy by FMDV to fine-tune infection through unique functional attributes residing within the La region of L protein. This regulatory mechanism further empowered FMDV to optimize its translation efficiency via the La gene, thereby bolstering its adaptability. Our research offered a foundational reference for deeper exploration into the specific roles of two L isoforms and the intricate translation initiation processes of FMDV.

## Figures and Tables

**Table 1:** Bioinformatics analysis of L protein sequence.

Prediction Type	Software Name	Prediction Result	Website URL
Signal peptide	SignalP 6.0 [24,25]	No classical secretion signal peptides	https://services.healthtech.dtu.dk/services/SignalP-6.0/ (accessed on 25 June 2025)
	IPSORT [26]	Residues 6–20 at the N-terminus were a potential non-classical secretion signal	https://ipsort.hgc.jp/ (accessed on 25 June 2025)
Nuclear localization signal (NLS)	NLStradamus r.9 [27]	No classical NLS	http://www.moseslab.csb.utoronto.ca/NLStradamus/ (accessed on 25 June 2025)
	cNLS Mapper [28,29]	No classical NLS	http://nls-mapper.iab.keio.ac.jp/cgi-bin/NLS_Mapper_y.cgi (accessed on 25 June 2025)
	DeepLoc 2.1 [30]	Residues 194–201 at the C-terminus were a potential non-classical NLS	https://services.healthtech.dtu.dk/services/DeepLoc-2.1/ (accessed on 25 June 2025)
Transmembrane α-helix (TMH)	TMHMM 2.0 [31,32]	No TMH	https://services.healthtech.dtu.dk/services/TMHMM-2.0/ (accessed on 25 June 2025)
	DeepTMHMM 1.0 [33]	No TMH	https://services.healthtech.dtu.dk/services/DeepTMHMM-1.0/ (accessed on 25 June 2025)
Subcellular localization	DeepLoc 2.1 [30]	Primarily localized to the nucleus (57.38%) and cytoplasm (54.36%)	https://services.healthtech.dtu.dk/services/DeepLoc-2.1/ (accessed on 25 June 2025)
	CELLO 2.0 [34]	Primarily localized to the cytoplasm (2.847), also detectable in the extracellular space (0.854)	https://cello.life.nctu.edu.tw/ (accessed on 25 June 2025)
	BaCelLo [35]	Detectable in the extracellular space	https://busca.biocomp.unibo.it/bacello/ (accessed on 25 June 2025)
	Euk-mPLoc 2.0 [36]	Localized to cytoplasm and extracellular	http://www.csbio.sjtu.edu.cn/bioinf/euk-multi-2/ (accessed on 25 June 2025)

## Data Availability

All data supporting the findings are available in this manuscript.

## Abbreviations

The following abbreviations are used in this manuscript:

FMDV	Foot-and-mouth disease virus
FMD	Foot-and-mouth disease
L	Leader
eIF4G	Eukaryotic translation initiation factor 4G
IRES	Internal ribosome entry site
IFN	Interferon
IFA	Immunofluorescence assay
CPE	Cytopathic effect
TCID50	50% tissue culture infective dose
NLS	Nuclear localization signal
SPs	Stress granules
LDs	Lipid droplets
MOI	Multiplicity of infection
BSA	Bovine serum albumin
DAPI	4′,6-diamidino-2-phenylindole
hpi	Hours post-infection
SDS-PAGE	Sodium dodecyl sulfate-polyacrylamide gel electrophoresis
GAPDH	Glyceraldehyde-3-phosphate dehydrogenase
